# 940. The use of a commercially available CMV T Cell Immunity Panel to Assess the Risk of CMV Infections in Hematopoietic Cell Transplant Recipients with Low Level CMV Viremia

**DOI:** 10.1093/ofid/ofab466.1135

**Published:** 2021-12-04

**Authors:** Fareed Khawaja, Carmen Sadaka, Samantha Trager, Kerri E Fernandes, Georgios Angelidakis, Ella Ariza Heredia, Michelle Altrich, Roy F Chemaly

**Affiliations:** 1 University of Texas MD Anderson Cancer Center, Houston, Texas; 2 MD Anderson Cancer Center, Houston, Texas; 3 : Departments of Infectious Diseases, Infection Control and Employee Health, Houston, Texas; 4 The University of Texas MD Anderson Cancer Center, Houston, TX; 5 eurofins Viracor, Lee’s Summit, Missouri

## Abstract

**Background:**

Cytomegalovirus (CMV) infections continue to be associated with increased morbidity and mortality in Hematopoietic Cell Transplant (HCT) recipients. Treatment of high risk patients with low level viremia may reduce overall duration of therapy and reduce complications. CMV T Cell Immunity Panel (TCIP) may help identify patients at high risk of CMV reactivation prior to developing clinically significant CMV infection (CS-CMVi). Our study aims to identify HCT recipients with low level CMV viremia who are at high risk of CMV reactivation with the use of CMV-TCIP while on or off letermovir for prophylaxis.

**Methods:**

We enrolled in a prospective cohort study allogeneic HCT recipients (excluding cord blood transplantation) with low level of CMV viremia (viral load of < 1000 IU/ml) on no therapy, starting October 2019. CMV TCIP assay was performed at enrollment, weeks 1, 2, 3, 4, 6 and 8. CMV TCIP results were interpreted as negative or positive based on percentage of interferon gamma producing CD4+ or CD8+ CMV specific T cells. The primary endpoint was progression to a CS-CMVi. We are presenting the results of the first 30 patients with data up to 4 weeks from enrollment.

**Results:**

Among the 30 patients, 73% were on letermovir for CMV prophylaxis. Majority of the patients were ≥ 40 years old (77%), male (63%), received transplant for AML (40%), were in complete remission at time of transplant (23%) and received cyclophosphamide (90%). The median time from transplant to enrollment was 77 days (IQR 37-172) (table 1). At enrollment, 10 (33%) patients had a positive CMV TCIP, 10 (33%) had a negative CMV TCIP, and 10 (33%) had an uninterpretable CMV TCIP result due to inability to quantify T cells (table 1). Four (13%) patients developed CS-CMVi; 3 of these patients had a negative TCIP and 1 had unquantifiable CMV TCIP (Figure 1). The mean percentage of CMV specific CD4+ and CD8+ interferon producing cells was 1.76% (SD 2.24) and 9.37% (SD 11.35) for those on letermovir and 2.09% (SD 2.05) and 3.97% (SD 5.24) off letermovir respectively (*P >0.05*) (Figure 2).

Figure 1. Breakdown of the 30 patients during the 4 week follow up period

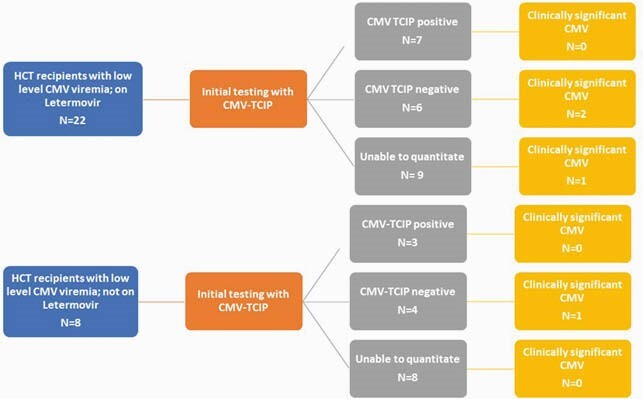

Abbreviations: HCT: Hematopoietic cell transplantation; CMV: Cytomegalovirus; TCIP: T cell immunity panel

Figure 2. Box-plot of percentage of CD4+ CMV specific interferon producing cells over time. Threshold for positive result (0.2%) marked.

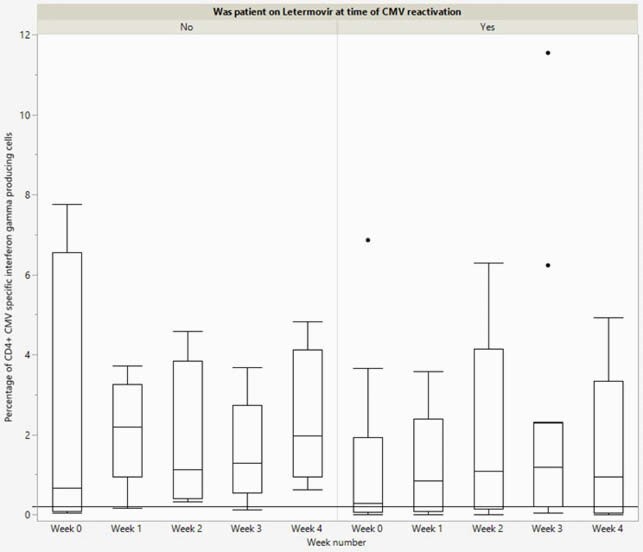

Abbreviations: CMV: Cytomegalovirus

Table 1. Baseline characteristics of patients enrolled

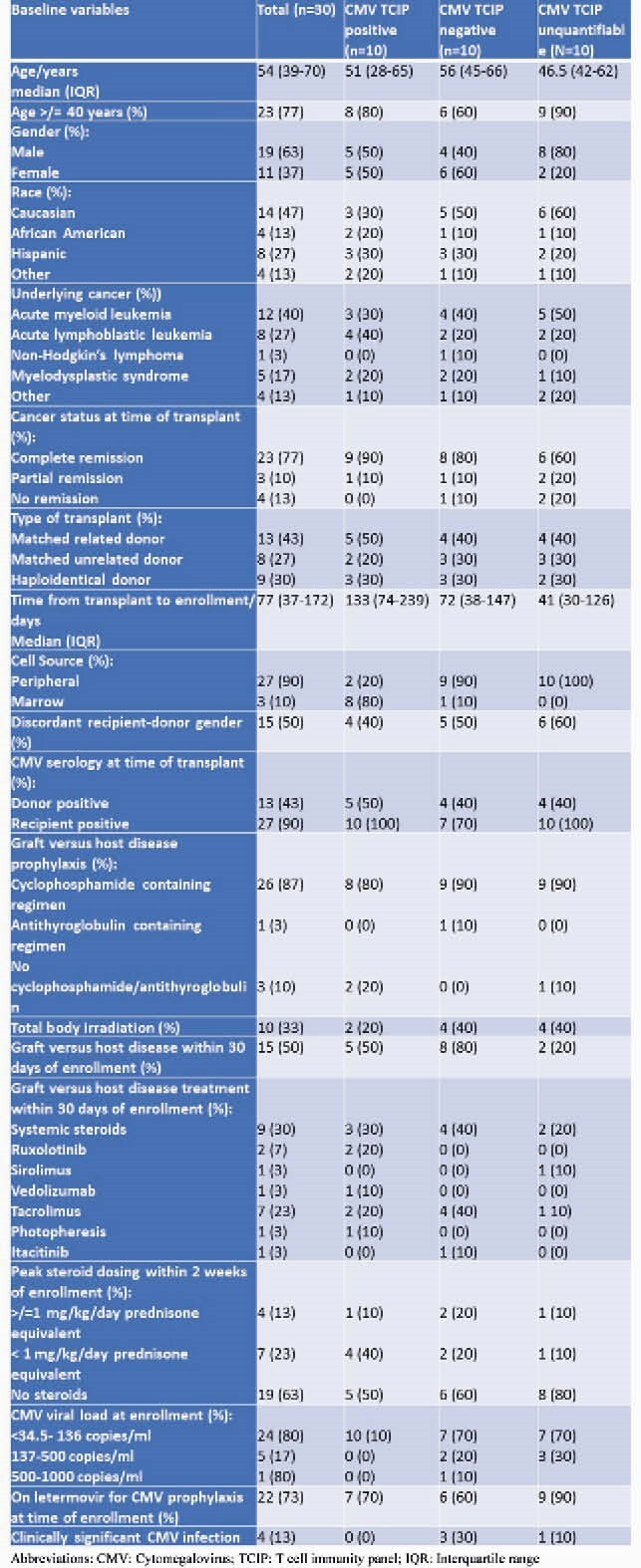

Abbreviations: CMV: Cytomegalovirus; TCIP: T cell immunity panel; IQR: Interquartile range

**Conclusion:**

Our results demonstrate the value of the CMV TCIP in identifying high risk HCT recipients prior to developing CS-CMV infection.

**Disclosures:**

**Fareed Khawaja, MBBS**, **Eurofins Viracor** (Research Grant or Support) **Ella Ariza Heredia, MD**, **Merck** (Grant/Research Support) **Michelle Altrich, PhD, HCLD**, **Eurofins Viracor** (Employee) **Roy F. Chemaly, MD, MPH, FACP, FIDSA**, **AiCuris** (Grant/Research Support)**Ansun Biopharma** (Consultant, Grant/Research Support)**Chimerix** (Consultant, Grant/Research Support)**Clinigen** (Consultant)**Genentech** (Consultant, Grant/Research Support)**Janssen** (Consultant, Grant/Research Support)**Karius** (Grant/Research Support)**Merck** (Consultant, Grant/Research Support)**Molecular Partners** (Consultant, Advisor or Review Panel member)**Novartis** (Grant/Research Support)**Oxford Immunotec** (Consultant, Grant/Research Support)**Partner Therapeutics** (Consultant)**Pulmotec** (Consultant, Grant/Research Support)**Shire/Takeda** (Consultant, Grant/Research Support)**Viracor** (Grant/Research Support)**Xenex** (Grant/Research Support)

